# Consucrats Have Agency: What Next for the Profecrat? Comment on "The Rise of the Consucrat"

**DOI:** 10.34172/ijhpm.2021.41

**Published:** 2021-05-01

**Authors:** Debbie Isobel Keeling

**Affiliations:** University of Sussex Business School, University of Sussex, Brighton, UK.

**Keywords:** Healthcare, Consumer Representation, Communities, Agency, Complex Services Marketing

## Abstract

The trend in ensuring adequate consumer representation across diverse activities and sectors, not least in healthcare, has been speedily implemented, sometimes at the expense of strategy. This commentary explores the concept of the consucrat as a consumer representative, presented by de Leeuw, which raised important questions regarding the way in which individuals and health services interact and collaborate. Adopting a complex services marketing lens, the position of the consucrat is discussed in relation to agency underpinning three tensions identified by de Leeuw: designation; professionalization, and; representation. For equality, professional service providers are referred to as ‘profecrats.’ Supporting de Leeuw, challenges are made to the underlying assumptions implicit in terms used around representation, the perspective that it is the consucrat only who needs to adapt, and the discourse around the competence of the consucrat. We should not be too cautious in our approach to consumer representation. Consucrats have agency – what next for the profecrat?

In ‘The Rise of the Consucrat,’ de Leeuw^1^ effectively sets out the ambiguity, contradiction and complexity of perspectives in the somewhat fuzzy rhetoric and implementation of consumer representation. Reflecting on government attempts to innovate consumer representation, de Leeuw^1^ offers the examples of femocrats and abocrats. The examples provided by de Leeuw present a sombre picture of how such designated representatives of specific communities are (in)conveniently labelled and sit (un)comfortably between community peers and government administration. Such initiatives do not easily or quickly achieve the desired shift towards enhanced respect of consumers as equals and knowledgeable representatives or provision of better service opportunities for the communities that they represent. This is because the assumptions underlying such role appointments neglect to account for the influence of the ‘individual’ in the role in terms of heterogeneity of motivations and ability to represent multiple views, in tandem with underlying structural or actual opportunities to be able to (or not) assert the consumer voice.^2,3^ In a similar vein, the concept of the consucrat is identified as representing the ‘volunteer channel of the voice of the receiving ends of healthcare’^1^ (p. 3). This representation places the consumer in a passive ‘receiving’ rather than an active ‘creator’ role. The position of the consucrat is rightly analysed by de Leeuw with regard to tensions relating to designation, professionalization and representation.

The tensions described by de Leeuw^1^ parallel those within the marketing domain, particularly the marketing of complex services. The study of complex services marketing recognises that complex services (eg, healthcare, legal, education and financial services) are highly person-orientated but underpinned by complex administrative and technical systems, are knowledge intensive with a reliance on professional expertise, and involve many actors working in an extensive service ecosystem with long service delivery times over multiple interactions. I apply this lens in my commentary as it offers valuable cross-disciplinary insights to the ongoing conversation. Much of the debate around consumer representation in complex services hinges on the notions of voice and agency. Despite the many stakeholder voices, there is no doubt that the service professional or provider voice is often the loudest.^4^ More fundamentally, labels assigned to individuals as stakeholders reveal the assumptions being made about the agency of those individuals. Debates in services marketing persist over the suitability of terms such as consumer, customer, service user, user, client, expert user, care-receiver, etc. At the heart of this debate is the way in which labels can promote passivity and demote active agency, by positioning the individual at the receiving end of service, eg, as ‘users.’^5^ As de Leeuw^1^ points out, representatives of communities are often referred to as ‘*crats.’ Whilst this suffix (literally meaning a person with power) should confer more power to those representatives, instead use of such terminology is reductionist and limiting as it is not applied universally to all actors. Rather its use serves to highlight the consucrat as an external role, not a fundamental part of the system. For equality, if consucrat is the label of the community representative, then perhaps we should refer to professional service representatives as ‘profecrats?’

Indeed, there is an ongoing debate in the domain of complex services marketing over the ‘role’ of consumers and professionals,^6-8^ which is closely aligned to the tensions raised by de Leeuw. In relation to the designation of agency, the nature of person-centred engagement within a multi-actor service eco-system is pertinent. With regard to the professionalization of agency, the power of and within dialogue to co-create shared understandings has relevance. Considering the representation of agency, a fundamental issue if the changing nature of stakeholder roles within multi-actor, multi-interactions over long service delivery periods.

## Designation of Agency

One tension arises from the designated role of the consucrat.^1^ As a designated ‘voluntary’ representative the consucrat is retro-fitted into the healthcare governance system. That is, they are added onto an existing system, rather than being a fundamentally essential part of that system or, indeed, the system being redesigned with their integration. Their roles are immediately bounded with respect to the nature and parameters of their engagement as written in the (organization’s) terms of reference. Where there is a prevailing assumption that the value of the consucrat’s input is limited to procedural and/or operational dimensions. Yet, individual agency is more than use of a service, or consent or compliance at the point-of-care. There is a need for meaningful recognition of the agency of the individual that sits not just within but also outside of the service being provided.^8^ Marketing scholars identify that a key challenge in complex health services is integrating individual agency right at the conception of healthcare design. Whilst achieving this is demanding, there is a danger in overlooking agency, especially of individuals within vulnerable or marginalised communities, leading to inadequate policy, poor service design, fragmented service experience, failure of innovations and further disenfranchisement of these communities.^9-11^

From a complex services marketing perspective, in designating the role of consucrat, we are moving away from terms such as participation, involvement, or even, designation. These terms focus on specific levels of activity and/or allocated resources rather than long term meaningful interactions. Hence, their use can preserve power imbalances and imply that ownership or responsibility for health and care direction and outcomes does not, at least partly, lie with the consucrat and that their input is assumed rather than enabled. Yet, from a marketing perspective it is increasingly recognised that meaningful negotiation between consumer and professional promotes mutual understanding, addresses power imbalances and, thus, is a pathway towards empowerment and engagement,^8^ but is not often universally practiced.^12^ What is needed is a deeper understanding of the meaning of health and care from the consucrat’s perspective. Such meaning can be quite distinct from the profecrat’s understandings, and can change substantially over the course of an individual’s care journey.

The medical view of value often depends on defined instrumentally distinct and measureable outcomes. Yet, such outcomes may not hold the same value for the consumer. For example, rather than physical recovery they may value preservation of social identity, even at the cost of their physical health.^13^ Further, agency is fluid and can be defined differently at each point on this journey, with respect to choices made or not made, level of desired engagement, and the allocation of their resources.^13^ In recognition of this, the rules of engagement should not be designated or dictated by organizational terms of reference. Engagement cannot be assumed; individuals can and do have good reasons to be unengaged with formal services.^5^ That is, it is important to recognize that consucrat agency operates outside of formal services. The greater accessibility of resources for and the utilization of self-service healthcare has the potential to disrupt, challenge or even replace formal care services. This is equally relevant to the often neglected agency of the informal carer.^4^ The profecrat can equally be unengaged with the consucrat’s journey with consequences for neglecting the value of health and care from the consucrat’s perspective, but this is rarely debated. The implication is that the consucrat, rather than being subject to the rules of engagement, should be at the heart of the continuous evaluation and development of such rules. From a services ecosystem perspective,^14^ enabling this would demand changes from the micro-level consultation upwards, upskilling consumers and professionals alike in challenging legacy assumptions in healthcare – not an insignificant task that requires a thorough understanding of dialogue and roles as explored in the next two sections.

## Professionalization of Agency

A second tension relates to the need for consucrats to master the professional rules of exchange and interaction.^1^ They need to somehow learn to reframe experience in professional language based on the assumption that this will provide more valuable input. As de Leeuw notes, they need to move from the ‘language of the street in order to engage with the language of the system’^1^ (p. 3). Yet, it is quite clear that elements of the consucrat’s perspective will be lost in translation. In agreement with de Leeuw, we should question the need for such professionalization of dialogue, as we do not see a dominant debate calling for the profecrat to abandon the language of the system for the language of the street. It simply persists in giving primacy to the professional service, not the individual journey. A more balanced approach would be to enable consumers and professionals to come to appreciate each other’s language.

Indeed, an alternative perspective, now developing within marketing, is to see tensions as crucial in the dialogue between stakeholders.^15^ That is, between the consucrat and profecrat there is an opportunity to co-create a shared understanding of healthcare and their roles within it by articulating and working through the underlying tensions. Such opportunities occur on a daily basis during micro-level consultations, but are not always maximized due to service (time) pressures, prevailing precedence of practice, and lack of ability or reluctance from either the consumer and/or the professional.^13^ The dialogic mechanisms used during these interactions plays a fundamental role in either enabling or disabling the resolution of tensions.^13^ Within healthcare the tensions of power, legitimacy of perspective and socio-emotional positions can be resolved through dialogic mechanisms that integrate within discussion consucrat and profecrat priorities (eg, with respect to outcomes), concerns (eg, the nature of risk) and experiences (eg, the lived journey).^13^ There is evidence that within this co-creation process of resolution of tensions, individuals can achieve a powerful and ‘professionalized’ view of healthcare services, being able to engage not just operationally or procedurally but also conceptually without abandoning their own credibility as an individual^13^. Indeed, not engaging in such co-creation can lead to a route of co-destruction for all stakeholders.^13^ At its ‘worst’ conclusion, this can lead to a withdrawal, either physically or emotionally, from the healthcare service with strong negative emotional consequences for the consumer.^13^

Mastering professional rules of exchange and interaction at the expense of the ‘language of the street’ can also seriously diminish the potential value of the service journey to both the consucrat and profecrat in terms of the value of experience on the journey and the outcomes of that journey. Instead, recognizing that at the outset consumers and professionals may have very different views on the value of healthcare, a co-created journey enables value to evolve dynamically over time and to present the opportunity for value to be shared between the consucrat and the profecrat. Indeed, the complex service journey with multiple stakeholders over multiple interactions arguably presents more opportunity for the co-creation of value than other services. But in the short term it demands conscious effort from all sides if in the long term such interactions are to become the norm and permeate through all levels (micro, meso, macro) of the ecosystem. The value potential extends beyond ‘successful’ physical health to include mutual respect with long reaching consequences for future service development.^13^ For consumers and professionals alike, shared values can enable multiple positive outcomes such as a validation of healthcare management, better insight into the choices and risks relating to treatment, and articulation of the socio-emotional vulnerabilities related to health. The outcome can be better quality dialogue and a strong, mutually respectful relationship between stakeholders at an operational level.^13^ This provides a basis for the development of more ‘positive’ policy as well as practice, such that, learning from the experience of co-creation can inform the development of policies that sensitively set-out how consumers and professionals can engage more fully on a co-created journey, with a need to recognize the heterogeneity and broad scope of representation of agency.

## Representation of Agency

With the third tension relating to representation^1^, we come full-circle to the initial challenge posed. Representation, or ‘true’ representation is challenging and often not fully achieved. In assigning individuals to the role of consucrats, there can be issues with descriptive representation (ie, to what degree the consucrat shares relevant characteristics with those they are selected to represent) and/or substantive representation (to what degree the consucrat represents the true interests of those that they are selected to represent), alongside the privileging of technical competence over experiential competence.^2^ Moreover, as de Leeuw^1^ points out, including a consucrat, for example, on a board of advisers, is not always an authentic attempt to achieve full representation – it can be a check-box exercise. Yet, consucrat representation has the potential to mitigate against fracturing of health services as tensions are exposed, articulated and addressed.^16^ When tensions relating to multiple agendas are not addressed, consumers and professionals can have “diametrically opposed views about the ideal structure of the service system”^16^ (p. 2260), leading to a fragmentation of service delivery as stakeholders move along divergent pathways. Addressing tensions through representation of diverse stakeholder views enables stakeholders to remain on the same service journey.^13^

A consideration of roles is pertinent here. The consucrat is also faced with managing the complex interface between the ‘street’ and the ‘institution.’ ‘You treat it: I live it!’ But is this their sole responsibility or even within their ability? From a complex services marketing perspective,^7^ all stakeholders have a responsibility to manage the complex interfaces inherent in healthcare. Not all do so, are able to do so or even want to do so.^17^ Further, the nature of health interactions are changing to incorporate ‘third’ and ‘virtual’ voices (eg, carers), with concomitant changes in agency.^4^ The more traditional view of the consucrat may be as having an ‘enhancement role’ where representation equals the exchange of information about the community that they represent to the professional community. This exchange enhances the professional’s role in directing care. Or as having an ‘empowerment role’ where representation equals contributing to discussion about treatment options from an informed perspective. Both roles can be enabled by current organizational infrastructure and processes.^18^ But, arguably, the most powerful is an ‘emancipation role’ where the consucrat acts as an independent challenger of the normalized service and profecrat assumed ‘knowledge’ of the community being represented^4^. Adopting this role can lead to the consucrat holding the profecrat to account, but not in a way that erodes the relationship, but one that constructively builds trust between both parties.^4^

Conceivably such a role cannot be readily carried out by one person, instead from a marketing view we can flip the perspective to look at the role of the profecrat within the community that consucrats reside. There is ample evidence that communities do challenge formal services. For example, the proliferation of online community care services offered by and to individual members of those communities. These ‘virtual services’ comprise a structured ecosystem that offer members a decentralized and flexible service outside of the usual professional service boundaries, increased access and to information and reduction of asymmetries, and a community-owned, co-created ‘knowledge and experience store.’ Members who engage in such communities gain confidence in managing their own health journey and report contributing more effectively (from their perspective) in formal service encounters.^4^ These virtual services represent a collective voice. That is, rather than comprising an uncontested single narrative around health and care, they evidence multiple informed debates and sophisticated self-moderation and regulation around the nature of those debates.^19^ Multiple views are given space but do not go undebated. They also enable significant capacity building (both in terms of knowledge and critical evaluation skills) within the communities that they serve. In some cases, such communities can offer rival, and credible, services to formally offered services,^19^ demonstrating the often undervalued proficiency of the consucrat and the communities that they represent. Yet, we also observe that engagement in such communities can bring about a deeper and more positive understanding of the service professional that can improve relationships informal service consultations.^8^ Indeed, there is evidence of profecrat engagement in and valuing of such communities, where their input is valuable in bringing about an understanding of the constraints on and experiences of service professionals as well as acting as an informal conduit to formal services.^19^

## Conclusion

De Leeuw’s perspective article raises important questions regarding the way in which individuals and complex health services do, can or could interact and collaborate. The trend to ensure that there is consucrat representation on boards, committees, and liaison groups, has sometimes been quickly implemented at the expense of strategy. That is, consideration is given to checking the box rather than strategically positioning the role of the consucrat (eg, what do they bring to the conversation? How can they challenge professional thought?), their ability to undertake that role and how to empower that ability. This has led to a distracting debate regarding consucrat competence. Distracting, first, as the focus has been on the consucrat rather than the organizational structures that can inhibit their roles, and, second and relatedly, as it has led to doubt around the potentially powerful role of consucrats. We should not be too cautious about emancipating the role of consucrats. At the same time, we should be cognisant of the broad scope and heterogeneity of representation and the pressures that exerts on individual consucrats as representatives. The ‘messy realities of public policy development’^1^ (p. 4) are often a consequence of a myopic approach, with poor representation of stakeholder views and unjustified lack of confidence in the non-professional. If we fully accept that consucrats have agency – then what is next for the profecrat? With an increasing evidence base of the value of co-creation, healthcare consumers continue to develop in knowledge, skills and roles, with support from increasingly sophisticated technologies and communities. The role of the professional equally changes, where we can envisage more understanding of the pressures and constraints that they operate under. A coming together of the lived experiences of the consucrat and the profecrat promises a more productive journey for all.^13^

## Ethical issues

 Not applicable.

## Competing interests

 Author declares that she has no competing interests.

## Author’s contribution

 DIK is the single author of the paper.
